# Effects of Subtypes of Child Maltreatment on CRP in Adulthood

**DOI:** 10.3389/fpsyt.2021.533722

**Published:** 2021-11-18

**Authors:** Vicki Bitsika, Christopher F. Sharpley, Mary E. McMillan, Emmanuel Jesulola, Linda L. Agnew

**Affiliations:** ^1^Brain-Behaviour Research Group, University of New England, Armidale, NSW, Australia; ^2^Belmont Hospital, Belmont, NSW, Australia

**Keywords:** maltreatment against children, abuse, CRP - C-reactive protein, depression, childhood

## Abstract

In order to evaluate the effects of specific forms of childhood maltreatment (CM) upon adult C-reactive protein (CRP) concentrations, and to further describe the potentially confounding role that recent life stress and depression hold in that relationship, 221 participants from rural Australia (*M* age = 44yr, SD = 17.8yr) completed self-report questionnaires and provided a blood sample. There were no sex differences in any variables across the 91 males and 130 females, but depression status did confound the association between global CM and CRP. The specific aspect of CM was identified as physical and mental health abuse, and this was significantly associated with CRP level in participants with depressive symptoms and those without. There was no significant confound from recent life stressors. Results hold implications for the diagnosis of CM-related CRP elevation and (potentially) depression.

## Introduction

### Childhood Maltreatment, Immunity, and Mental Illness

A great deal of research has reported on the association between childhood maltreatment (CM) and the later development of mental illness in adulthood (1, 2). Although the exact pathways between CM and adult mental illness are not yet completely clear, one that has received support is via the innate immune system, which provides a first line of defense against pathogens as well as contributing to the adaptive set of responses known as ‘sickness behavior' (3). These responses include lethargy, fatigue, poor sleep, decreased appetite, psychomotor retardation and cognitive impairment, which are also core diagnostic criteria for Major depressive Disorder (MDD) (4). Although these symptoms are observed in individuals who receive pro-inflammatory cytokines such as interferon-α (5), inflammatory signaling processes instigated by the innate immune system include a range of cytokines. One that has received a good deal of attention for its association with CM is C-reactive protein (CRP) (6).

### CM and C-Reactive Protein

CRP is a major acute-phase plasma protein that recognizes altered and foreign molecules and binds to various ligands to play a role in the innate immune system and to stimulate phagocytosis (7, 8). In their longitudinal study of over 1,000 residents of Dunedin, NZ, (9) found that cumulative exposure to CM was significantly associated with elevated CRP 20 years later. CRP has also been found to be elevated in patients with a range of psychiatric disorders, including MDD (10, 11), an association that is not confounded by adult stress, poor adult health, unhealthy behavior patterns, or acute infections, as confirmed in over 30 studies [see (12), for a review]. However, there are also non-confirmatory findings regarding the link between CM and CRP. For example, (13) found no significant association between CM and adult CRP in 92 healthy adults, and other studies have found the association to be present only in patients with MDD (14) or treatment-resistant depressed patients (15). However, on average, meta-analytic reviews have argued for the presence of a CM-elevated CRP in adulthood association that is independent of age, gender, and body mass index (12), and that these two factors are also significantly associated with depression (16).

### CM, CRP, and Depression

Several details remain unclear regarding these overall associations between CM, CRP and depression. First, although the risk of other medical disorders (e.g., heart disease, cancer, lung disease, skeletal fractures, liver disease) appears to be directly related to the number and magnitude of CM experienced (16), similar results have yet to be reported regarding the frequency of CM and CRP and adult depression. Second, CM has been found to be significantly related to reactivity to subsequent psychosocial challenges (6) but not consistently associated with self-reports of recent life stressors (9). Third, the associations between specific types of CM and elevated adult CRP remain yet to be firmly established. For example, in their meta-analysis of 18 studies (16,870 individuals) for CM and CRP, (12) found “evidence that individual types of trauma exposure impact differentially on the inflammatory markers” (p. 647), with adult CRP elevation primarily related to parental absence rather than sexual abuse, physical abuse, or emotional abuse. Those authors commented that this differentiation between types of CM and CRP remained a major issue for research.

### Study Aims

Consequently, the following research questions were investigated in this study: (1) whether CRP was significantly and directly correlated with the frequency of CM (2) which type of CM was significantly associated with elevated CRP (3) whether recent life stressors (i.e., experienced as an adult) moderated the association between specific CM, or frequency of CM, and CRP, and (4) did depression status (i.e., presence of depressive symptoms at a defined level *vs* not at a defined level) moderate these associations In order to address these research questions from the widest possible base, a community sample was recruited. In addition, almost all of the studies reviewed above were performed on urban or city dwellers in large metropolitan centers. To extend those findings, the current study focussed upon rural residents in Australia. Although the prevalence of depression among Australian rural and city dwellers is comparable (17), the former resort to suicide more often than their depressed counterparts who live in cities (18), further justifying attention to this population.

## Methods

### Participants

Participants were volunteers from the New England region in rural New South Wales. The project was advertised by publicity and news media as “a study about mental health”. Inclusion criteria were that participants were to be at least 18 years of age. Exclusion criteria were the presence of an acute medical illness.

### Instruments

Background questionnaire. Participants were asked to state their age (years) and sex, and if they suffered from an acute mental illness that would impede their ability to respond accurately.

Childhood Stressors were measured via a dichotomous (present/absent) response format to 29 questions including the content and structure of those used in previous major studies (19) and the National Comorbidity Survey (20). Questions asked participants to state (‘yes' or ‘no') whether “any of the following happened to you when you were a child”, and included content regarding (but not limited to) parental marital separation and divorce; experiences of neglect; physical, sexual and mental abuse; parental unemployment; parental drug/alcohol abuse; suffering from a serious illness; mistreatment at school; family members' suicide or attempted suicide; death of a parent. This content argues for the validity of the scale, and the internal consistency (Cronbach alpha) of this scale in the current study was 0.96.

Recent Life Stressors were measured via dichotomous responses to the 14-item Recent Life Stress scale developed for the Hawaii Personality and Health Cohort Study (HPHCS), a population-based cohort participating in a longitudinal study of personality and health spanning 40 years from childhood to midlife (21). Participants were asked whether a series of stressful events had occurred to them during the last six months. These events included: death or serious injury of a parent, partner, child, close relative, relationship breakdown, serious problems with relatives, close friends or neighbors, being unemployed or losing a job, having major financial problems, difficulties with the police or law, and losing something valuable, supporting its content validity. Internal consistency for this scale in the current study was 0.89.

Depression was assessed by the 20-item Zung Self-rating Depression Scale (SDS) (22). The SDS is based on data from factor analytic studies of MDD (23) and fits the most recent definitions of that disorder (4). Respondents indicate the frequency of each of those 20 items by answering: “None or a little of the time”, “Some of the time”, “Good part of the time”, or “Most or all of the time”, which produce numerical scores of 1 to 4, providing total raw scores from 20 to 80. SDS raw scores of 40 or above indicate the presence of “clinically significant depression” based on validity studies with clinician's diagnoses (24), p. 335. Although this criterion does not equate to a formal diagnosis of MDD via professional clinical interview based on the DSM or ICD nomenclature, it was judged to be acceptable in this research study where such individual interviews were not logistically possible. To distinguish those participants who met the SDS criteria defined above, the terms “SDS-depressed” and SDS-non-depressed” were used. The SDS has demonstrated split-half reliability of 0.81 (22), 0.79 (25) and 0.94 (26). Internal consistency (alpha) has been reported as 0.88 for depressed patients and 0.93 for non-depressed patients (27) and as 0.84 for a previous Australian sample (28). The SDS has been shown to be superior to the MMPI Depression Scale and the Beck Depression Inventory for assessing depression in male psychiatric inpatients (27). SDS raw scores were used in this study. Cronbach's alpha for the SDS in this study was 0.775.

### CRP Assays

Blood samples were collected and centrifuged at 1000 g for 15 min. The sera were frozen at −80°C until analysis of C-reactive protein. Serum concentrations of CRP were determined using a Siemens Dimension XPand Plus Autoanalyser (Siemens, Newark, USA), using the CRP extended range (RCRP) Flex reagent cartridge (Siemens Dimension, Newark, USA) according to the manufacturers' instructions. This assay is based on the particle-enhanced turbidimetric immunoassay (PETIA) technique, where synthetic particles coated with anti-CRP antibodies aggregate in the presence of CRP, increasing turbidity in proportion to CRP concentration. Concentrations are reported in mg/L.

### Procedure

From a list of 20,000 random names and addresses (balanced for equal numbers of males and females) supplied by the Australian Electoral Commission in 2013, sufficient participants were recruited to exceed the sample size required by *a priori* power analysis for a correlational analysis to detect a ‘medium' effect of 0.3 or greater (29) with alpha = 0.05 and power = 0.95 (GPower 3.1). Participants received a link to an online portal or a copy of the questionnaire booklet containing an explanatory statement and consent form, plus the questionnaire booklet, and a request to attend the researchers' lab a few days later to provide a blood sample for CRP assay. CRP is relatively stable over time (30) and would not have varied during the brief period between questionnaire completion and serum collection. The project was approved by the University of New England Human Research Ethics Committee. All participants gave written consent to the study.

### Statistical Analyses

Data were tested for normality. Pearson correlation coefficients were calculated to test for any significant age effects on the DVs, and gender was also tested for its effect via MANOVA. Pearson correlation coefficients were calculated for the associations between the CRP and psychological variables, with a Bonferroni correction for multiple testing. Hierarchical regression tested for the relative effects of Recent Life Stressors on the association between CM and CRP.

## Results

### Data

A total sample of 221 participants who did not report suffering from an acute mental illness was recruited for this study (*M* age = 44.06yr, SD = 17.83yr, range = 18 to 85yr), including 91 males (41.2%) and 130 females (58.8%). CRP data were skewed and log transformed, but none of the psychological variable data were non-normal and so did not require transformation. Table 1 shows the mean, SD and range scores for all DVs. There were no significant correlations between age and CRP log, SDS, Recent Life Stress score or Childhood Stressors score, and there were no significant differences in any of these variables according to gender. Pearson correlation coefficients were calculated between variables and appear in Table 2. Allowing for the correction to reduce Type 1 error rates (i.e., 0.05/4 = 0.0125), there was a significant direct correlation between Childhood Stressors and SDS score.

**Table 1:** Distributions of all dependent variables.

**Variable**	**Mean**	**SD**	**Range**
SDS total score	37.5	9.2	21 to 66
Recent Life Stressors	26.7	1.2	23 to 28
Childhood Stressors	43.8	10.3	28 to 56
CRP mg/L	5.3	7.4	55 to 75.85
CRP log	0.58	0.32	−0.26 to 1.88

**Table 2:** Pearson correlations between variables.

**Variables**	**Age**	**SDS**	**Childhood stressors**	**Recent life stressors**
SDS	−0.141			
Childhood Stressors	0.062	0.343^*^		
Recent Life Stressors	0.039	0.181	0.187	
CRP log	−0.115	0.029	0.122	0.020

* *p < 0.0125*.

### Childhood Stressors and CRP

#### Frequency of CM

Whether frequency of CM was associated with CRP log for the entire sample was tested by the correlation coefficient between CM and CRP log, which was not significant at the corrected level (Table 2). However, when the sample was divided according to their SDS total score at the cutoff point of a raw score of 40 recommended by the SDS author (24), there was a significant correlation between CM and CRP log for the 90 participants in the ‘SDS-depressed' subsample (*r* = 0.274, *p* = 0.009) but not for those 130 participants who did not reach this cutoff score (*r* = 0.155, *p* = 0.076).

#### Type of CM

As mentioned above (Methods) the 29 Childhood Stressors comprised a heterogeneous mix of different types of events. To identify any associations dependent upon the types of CM that were described by (12), these 29 CM events were grouped into four subscales according to whether they referred to the child's own direct experiences (e.g., being verbally or physically abused, humiliated, harassed, or suffered mental cruelty), the child's parents' experiences (e.g., parents had alcohol, drug or mental health problems, were separated or divorced, or were prisoners), the experiences the child had at school or from other adults (e.g., mistreatment from a teacher or from other children at school) and the child's family's experiences (e.g., family members suicided, died, or the child witnessed abuse of siblings). Of these, only the child's own direct experiences were significantly correlated with CRP (*r* = 0.203, *p* = 0.020). Further exploration of the seven childhood stressors that comprised this subscale revealed that only those events which were directly related to the child's experiences of physical and mental abuse (at the adjusted *p* value of 0.05/7 = 0.0071) were significantly correlated with CRP (Table 3). When combined into a new variable, these two childhood stressors were significantly and strongly correlated with CRP (*r* = 0.337, *p* = 0.00008) and SDS total score (*r* = 0.292, *p* = 0.0007) in the total sample, and also within the SDS-non-depressed (CRP: *r* = 0.233, *p* = 0.007; SDS: *r* = 0.258, *p* = 0.003) and SDS-depressed (CRP: *r* = 0.332, *p* = 0.001; SDS: *r* = 0.242, *p* = 0.022) subsamples.

**Table 3:** Pearson correlations between CRP and child-direct childhood stressors.

**Variables**	**Neglected**	**Verbally abused by parents**	**Humiliated, harassed, mentally cruelty**	**Physically abused**	**Excessive physical punishment**	**Mistreatment from an adult (non-family)**	**Sexually abused**
CRP log	0.130	0.103	0.258^*^	0.242^*^	0.107	0.053	0.126
SDS score	0.366^*^	0.260^*^	0.269^*^	0.216	0.102	0.222	0.168

* *p < 0.007*.

#### Effect of Recent Life Stress

In the total sample, adult CRP was significantly predicted by the combined physical and mental abuse childhood stressor [R square = 0.114, *F*_(1,129)_ for change = 16.676, *p* = 0.00007] but the addition of Recent Life Stress to the hierarchical regression equation did not produce a significant increase in the variance explained by the combined physical and mental abuse variable [R square change = 0.006, *F*_(1,128)_ = 0.874, *p* = 0.351] and neither did the addition of the SDS total score [R square change = 0.004, *F*_(1,127)_ = 0.562, *p* = 0.351]. These findings were consistent across the SDS-depressed and SDS-non-depressed subsamples.

## Discussion

### Findings

The major finding from this study is that the association between childhood stressors and adult inflammatory status was a function of particular childhood experiences relating to being verbally and physically abused, and was not significantly altered by recent stressful events, nor whether the participants were SDS-depressed or not. In terms of the first research question posed in the Introduction, frequency of CM was directly and significantly correlated with CRP, but only for those participants who met the SDS cutoff score for “clinically significant depression” (24) p. 335, arguing for an interaction between overall CM and CRP and depression status. That this association was present for only SDS-depressed participants argues either for consideration of a vulnerability-to-depression effect for the association between overall CM and CRP, or for the existence of the CM-CRP link to be an outcome of depression. At present, and without longitudinal data, this directionality cannot be determined.

One particular aspect of CM was identified in this association—that of the child's experience of physical and mental health abuse. This aspect was powerful enough to exist for SDS-depressed and SDS-non-depressed participants, suggesting that it was at least somewhat independent of depression status *per se*. The lack of any significant association between CRP and other aspects of CM (such as sexual abuse, parent misfortune, family problems, school-based issues) appears to highlight one aspect of the negative experiences of children and its association with their later inflammatory state, whether they are SDS-depressed or not.

The third research question addressed the effect of recent life stress upon the association between childhood physical and mental health abuse and CRP, and results indicated that the total score from the Recent Life Stress inventory did not make a significant contribution to the variance in the relationship between physical and mental health abuse during childhood and CRP in adulthood for either SDS-depressed or SDS-non-depressed participants. Finally, depression status had an intermittent effect upon only the global CM-CRP association, influencing the association for the SDS-depressed participants but not the SDS-non-depressed participants.

### Implications for Assessment and Treatment

Although any implications for assessment and treatment are only suggestive at this stage, and require further investigation and evaluation of the processes described below, these findings have the potential to advance understanding of the ways that CM interacts with depression to influence CRP in adulthood by detailing which specific aspect of CM was influential in the association between CM and CRP. This information has the potential to assist in screening those adults whose CRP may be elevated (and hence at greater risk of several mental and physical illnesses) as an outcome of their CM. As an example that requires further empirical evaluation, treatment protocols for persons with this CM-CRP link may require different foci than those where elevated CRP is not associated with CM that included mental and physical abuse. The former might benefit from psychotherapy that deals with childhood experiences, an approach which has been found to be effective in treating depression in adults (31), whereas those patients whose CRP is independent of their CM might be more appropriately treated with psychotherapies that focus on “here-and-now” problem-solving (32). Although depressed patients with elevated inflammation are less likely to respond to standard medications for depression (33), the application of adjunctive anti-inflammatory treatments has been shown to be effective (34). These comments are hesitant at this stage because CM is associated with alterations in various important processes, and so it remains a highly relevant issue when diagnosing depression, even when CRP variations are absent.

### Limitations

This sample was restricted in terms of geographical and cultural region, and extension of these findings to other cultures and nations would clarify the generalisability of the results. That limitation also applies to the fact that, although these data were purposely collected from a rural sample, no direct comparison was able to be made with an urban sample. Although it is a characteristic of research designs such as this one, the data are not generalizable over time or circumstance, and longitudinal data-collection would assist in identifying any temporally-based causal connections. The classification of CM into the four subgroups was done on the basis of the source of child stress, but other classifications could be applied and may reveal different results. CRP is relatively stable, and may be regarded as not reasonably differing in the time between blood collection and completion of psychological tests. The measure of childhood events is demonstrably valid, but does not measure the severity of the stressor event, which could be included in future research. Reducing data from a continuous scale such as the SDS into a dichotomous variable always reduces the power of the analysis (35). Depressive symptoms may exhibit variation across individuals, and these variations could also be related to CRP. The use of a standardized self-report scale for depressive symptoms does not provide a clinician-based assessment using DSM or ICD criteria, and that methodology would add verisimilitude to the data. Although all participants were screened on the basis of having an acute medical illness (see section Participants), no formal examination of participants was undertaken and it may be that some health comorbidity may have influenced CRP levels. Self-selection may bias the respondent sample in surveys like this one, and is a possible limitation. However, when surveys such as this are undertaken for the purpose of obtaining a random community sample, little can be done to obviate such bias except to acknowledge its possible presence. Strengths of this study are use of well-validated scales for measuring depression recent stressors, the sample size, and the extension of previous research into a rural setting. Future studies might undertake mediation and moderation investigations of this kind of data but this study was purposely restricted to correlational and regression analyses as a first exploratory step.

## Conclusions

These data support the previously-reported association between CM and CRP, but extend those findings by defining which aspects of CM were most strongly associated with CRP in adulthood. The lack of any significant effects due to recent stress was also provided in a more detailed manner, and the contributory role of depression was also clarified. Implications that these data have for diagnosis and treatment were explained.

## Data Availability Statement

The datasets generated for this study are available on request to the corresponding author.

## Ethics Statement

The studies involving human participants were reviewed and approved by Human Subjects Research Ethics Committee, University of New England. The patients/participants provided their written informed consent to participate in this study.

## Author Contributions

All authors listed have made a substantial, direct, and intellectual contribution to the work and approved it for publication.

## Conflict of Interest

The authors declare that the research was conducted in the absence of any commercial or financial relationships that could be construed as a potential conflict of interest.

## Publisher's Note

All claims expressed in this article are solely those of the authors and do not necessarily represent those of their affiliated organizations, or those of the publisher, the editors and the reviewers. Any product that may be evaluated in this article, or claim that may be made by its manufacturer, is not guaranteed or endorsed by the publisher.
